# 3D Single-Breath Chemical Shift Imaging Hyperpolarized Xe-129 MRI of Healthy, CF, IPF, and COPD Subjects

**DOI:** 10.3390/tomography8050215

**Published:** 2022-10-13

**Authors:** Steven Guan, Nick Tustison, Kun Qing, Yun Michael Shim, John Mugler, Talissa Altes, Dana Albon, Deborah Froh, Borna Mehrad, James Patrie, Alan Ropp, Braden Miller, Jill Nehrbas, Jaime Mata

**Affiliations:** 1 Department of Radiology and Medical Imaging, University of Virginia, Charlottesville, VA 22903, USA; 2 Department of Radiation Oncology, City of Hope, Duarte, CA 91010, USA; 3 Department of Medicine, University of Virginia, Charlottesville, VA 22903, USA; 4 Department of Medicine, University of Missouri, Columbia, MO 65211, USA; 5 Department of Medicine, University of Florida, Gainesville, FL 32611, USA; 6 Department of Public Health, University of Virginia, Charlottesville, VA 22903, USA

**Keywords:** hyperpolarized xenon-129, MRI, COPD, cystic fibrosis, idiopathic pulmonary fibrosis

## Abstract

3D Single-breath Chemical Shift Imaging (3D-SBCSI) is a hybrid MR-spectroscopic imaging modality that uses hyperpolarized xenon-129 gas (Xe-129) to differentiate lung diseases by probing functional characteristics. This study tests the efficacy of 3D-SBCSI in differentiating physiology among pulmonary diseases. A total of 45 subjects—16 healthy, 11 idiopathic pulmonary fibrosis (IPF), 13 cystic fibrosis (CF), and 5 chronic obstructive pulmonary disease (COPD)—were given 1/3 forced vital capacity (FVC) of hyperpolarized Xe-129, inhaled for a ~7 s MRI acquisition. Proton, Xe-129 ventilation, and 3D-SBCSI images were acquired with separate breath-holds using a radiofrequency chest coil tuned to Xe-129. The Xe-129 spectrum was analyzed in each lung voxel for ratios of spectroscopic peaks, chemical shifts, and T2* relaxation. CF and COPD subjects had significantly more ventilation defects than IPF and healthy subjects, which correlated with FEV1 predicted (R = −0.74). FEV1 predicted correlated well with RBC/Gas ratio (R = 0.67). COPD and IPF had significantly higher Tissue/RBC ratios than other subjects, longer RBC T2* relaxation times, and greater RBC chemical shifts. CF subjects had more ventilation defects than healthy subjects, elevated Tissue/RBC ratio, shorter Tissue T2* relaxation, and greater RBC chemical shift. 3D-SBCSI may be helpful in the detection and characterization of pulmonary disease, following treatment efficacy, and predicting disease outcomes.

## 1. Introduction

Nearly 500 million people throughout the world are directly affected by respiratory diseases [1]. Different respiratory diseases are classified by either the organs affected or based on whether they are obstructive or restrictive. In obstructive pulmonary diseases, lung defects persistently hinder airflow into and out of the lungs. Two common obstructive lung diseases include chronic obstructive pulmonary disease (COPD) and cystic fibrosis (CF). In restrictive lung diseases, there is a reduction in lung volume, either because of changes in the lung parenchyma or alterations leading to difficulty expanding the chest wall during inhalation. One example of a common restrictive lung disease is idiopathic pulmonary fibrosis (IPF).

COPD, CF, and IPF are all progressive in nature. COPD is a tissue disease associated with genetic risk factors that is primarily caused by long-term exposure to substances such as cigarette smoke or air pollution [2]. Meanwhile, CF is a genetic disease characterized by airway obstruction that results from abnormally thick mucus [3]. IPF is a chronic, progressive interstitial lung disease in which there is enhanced extracellular matrix deposition. In most cases, IPF is triggered by injury or long-term exposure to hazardous chemicals causing lung tissue to become scarred and thickened [4]. This reduces lung volume and restricts the diffusion capacity for carbon dioxide (DLCO) as well as the amount of oxygen that can pass into the blood [5].

As progressive diseases, COPD, CF, and IPF require persistent monitoring to ensure patients are receiving the appropriate treatment as their lungs change with time. Additionally, these diseases present with many phenotypes and are often accompanied by comorbid diseases [6,7,8]. Due to the widely varying nature of COPD, CF, and IPF and the tendency for comorbid disease, it is important to have a single method that can screen subjects, monitor disease progression, and characterize the influence of co-morbidities. Currently, computed tomography (CT) is used to image patients’ lungs and determine disease severity. While CT is high resolution and can provide visualization of anatomical changes and defects in the lungs, it exposes patients to radiation and does not provide direct information about functionality such as ventilation or gas exchange. Since the lungs are one of the most sensitive organs in the body to radiation, repetitive scans are damaging and increase patients’ risk of developing radiation-induced cancer [9,10]. Spirometry, another common pulmonary assessment, only provides information about global pulmonary function. Thus, it is critical to develop new imaging techniques for COPD, CF, and IPF patients that have reduced risks and provide more detailed regional information. 

To address this, we have developed a non-invasive, nonradioactive imaging method which uses 3D Single-breath Chemical Shift Imaging (3D-SBCSI) and hyperpolarized Xe-129 [11,12,13]. When inhaled, hyperpolarized Xe-129 has the property of dissolving into the lung tissue and from there binds to the red-blood-cells (RBC). Because of this property, it can be used has a probe for each phase of gas dissolution in the lung (airway, tissue, and blood) because it has three distinct corresponding spectral peaks [14,15]. By detecting and measuring these peaks, we can exploit xenon’s natural sensitivity to its chemical environment and assess the lungs based on these chemical shifts [16,17]. In a rat model, researchers showed that 3D-MRI with hyperpolarized Xe-129 is sensitive to impairments in gas exchange caused by fibrotic thickening [18]. When translated to human studies, hyperpolarized Xe-129 has been used to assess regional ventilation and gas exchange in lung tissue and RBCs of smokers and cystic fibrosis patients [19,20,21]. Importantly, 3D-SBCSI patients are not subjected to radiation, allowing patients to have many scans without the risk of radiation-induced complications [22]. Xe-129 is also abundant in the atmosphere, not very expensive, and has been shown to be very sensitive to ventilation abnormalities [22,23,24]. Other methods to probe Xe-129 gas exchange, like the 1-point Dixon, 2-point Dixon and 3-point Dixon acquisitions may offer greater speed and resolution, but 3D-SBCSI generates the full spectrum of peaks per voxel and thus provides much more information [25,26]. In this study, we explore 3D-SBCSI with hyperpolarized Xe-129 in healthy, CF, IPF, and COPD subjects to elucidate differences among these pulmonary disease types and to correlate these findings with spirometry.

## 2. Materials and Methods

A total of 45 subjects participated in this study: 16 healthy (28 ± 9.8 years old), 11 with IPF (66 ± 11.6 years old), 13 with CF (24 ± 8.7 years old), and 5 with COPD (64 ± 11.8 years old). Within the CF population, nine were categorized as mild (FEV1% > 60) and four as moderate (FEV1% < 60). Each subject was imaged in a 1.5T MR scanner (Avanto, Siemens Medical Solutions, Malvern, PA, USA), using a commercial RF coil (Clinical MR Solutions, Brookfield, WI, USA) tuned to the Xe-129 frequency and underwent spirometry testing before the MRI. Written informed consent was obtained from all subjects, and the study was performed under a protocol approved by the Institutional Review Board at the University of Virginia. Healthy and CF subjects were imaged twice for repeatability.

All imaging was performed using a transmit/receive RF chest coil tuned to the frequency of Xe-129. The subjects laid supine on the MR table and inhaled a volume of gas mixture equal to 1/3 of their FVC, with a total maximum volume capped at 1000 mL of isotopically enriched (83%) Xe-129 mixed with nitrogen. Xe-129 was polarized to ~35% using a commercial polarizer (Polarean, Durham, NC, USA). Proton, ventilation, and 3D-SBCSI images were acquired for each subject. Subjects held their breath for less than 10 s during the imaging sequence, which proton (2D-GRE sequence with spiral trajectories; TA < 2 s) and either 3D-SBCSI (TA ~7 s) or ventilation images (TA ~2.7 s) were acquired. This allowed each 3D-SBCSI or ventilation image slice to be matched with a proton image from the same breath-hold. MR imaging sequence parameters for the 3D-SBCSI were: TR 13 ms; TE 1.0 ms; FA 25° centered at 200 ppm of the gas frequency; vector size 512; weighted phase-encoding; BW 50 kHz; minimum voxel size 6.5 × 6.5 mm^2^ and 6–8 slices with 20–25 mm slice thickness. For ventilation acquisition we used a 2D-GRE sequence with spiral trajectories and the following parameters: TR 11.4 ms; TE 1.19 ms; FA 20° centered at the gas frequency; 12 interleaves; total acquisition time for 17 slices was 2.7 s.

3D-SBCSI images were post-processed in MATLAB (Natick, MA, USA) using a software package developed in-house that analyzes the Xe-129 spectrum for each lung voxel in the 3D-SBCSI image. The free-induction decay signal (FID) was zero-filled from 512 to 1024 data points, apodized with a 50 Hz Lorentzian filter, Fourier transformed, and phase-corrected. Given that the three peaks (Xe-129 in gas, tissue, and RBC) overlapped, the spectrum was fitted to a sum of complex Lorentzian functions and optimized with a nonlinear least-squares algorithm. Finally, the ratios of spectroscopic peaks, chemical shifts, and T2* relaxation times were calculated on a voxel-by-voxel basis. Maps showing the computed parameters were then generated.

Ventilation images were segmented into regions of no ventilation, hypoventilation, normal ventilation, and hyperventilation using Advanced Normalization Tools software package (ANTs) [27,28] and fused with proton images in ITK-SNAP [29]. We used a N4 bias field correction, a whole lung segmentation and a Gaussian mixture model with a Markov random field spatial prior modeling [30]. Regions of no ventilation and hypoventilation were grouped as ventilation defects (VD); regions of normal ventilation and hyperventilation were grouped as healthy/normal. Whole-lung averages were computed for each parameter for each disease, and results were analyzed in MATLAB using one-way ANOVA and Tukey’s test for post hoc analysis.

## 3. Results

### 3.1. Comparison of Ventilation Images

**Figure 1:**
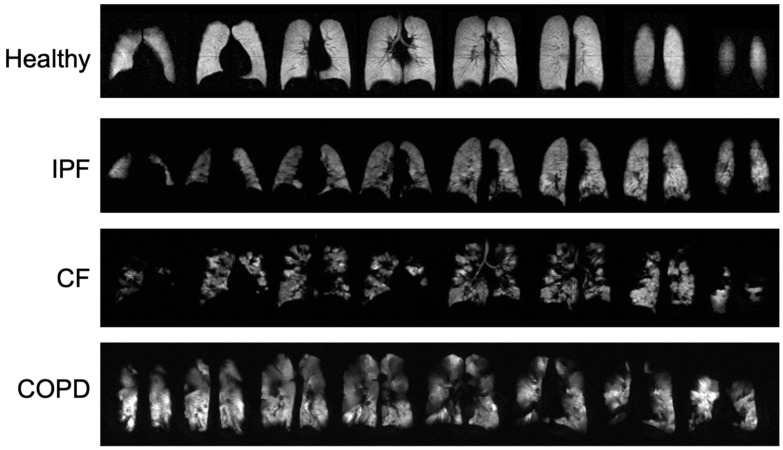
Ventilation images from a healthy subject and each disease type. Bright, homogeneous areas show that the lungs are ventilating normally.

**Figure 2:**
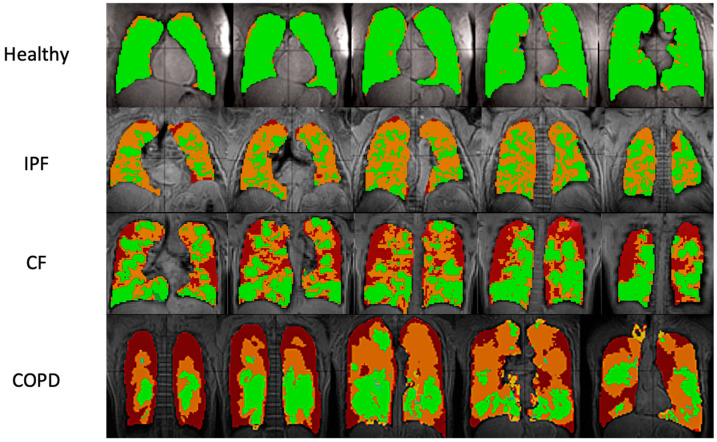
Ventilation images overlaid on proton images in sample healthy, IPF, CF, and COPD subjects. Each row shows five lung slices from anterior (**left**) to posterior (**right**). Green areas indicate regions of normal ventilation and hyperventilation. Orange areas indicate regions of hypoventilation. Red areas indicate regions of no ventilation.

Typical ventilation images of each disease are shown in Figure 1, and segmented ventilation maps are shown in Figure 2 (Figure 1 and Figure 2). IPF subjects had 28.1 ± 6.44 percent of lung volume occupied by ventilation defects (%VD), CF subjects had 39.1 ± 13.86 %VD, COPD subjects had 59.4 ± 9.14 %VD, and healthy subjects had 9.6 ± 7.37 %VD (Table 1). CF and COPD both had significantly more ventilation defects than IPF (*p* < 0.05) and healthy subjects (*p* < 0.001); CF and COPD subjects also had more regions of no ventilation than IPF and healthy subjects (*p* < 0.01). IPF had more ventilation defects than healthy subjects (*p* < 0.001). COPD subjects also had more defects than CF subjects (*p* < 0.01). %VD corresponded strongly with FEV1 predicted (R *=* −0.74).

### 3.2. Comparison of Peak Ratios

**Figure 3:**
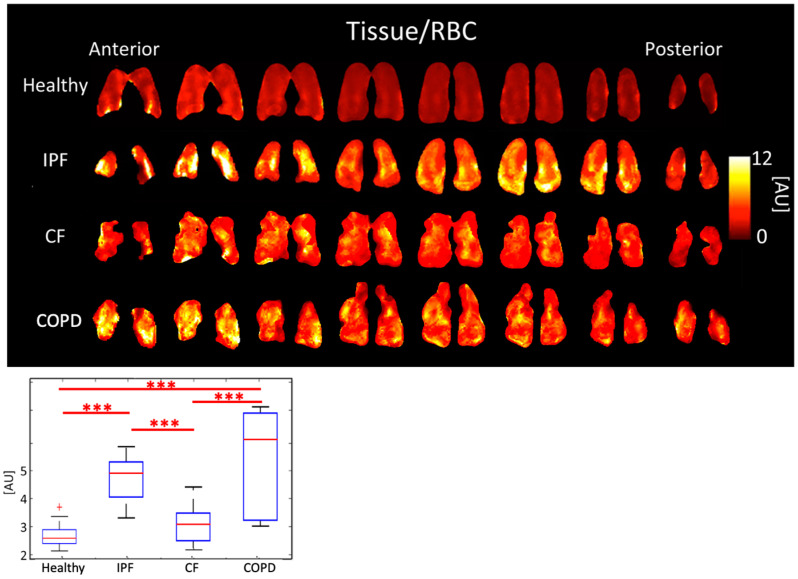
Tissue/RBC ratio maps (**top**) and boxplots (**bottom**) for each disease type. (*p* < 0.001 *=* ***, ^+^ outlier).

IPF and COPD had the highest Tissue/RBC peak ratios of 4.71 ± 0.807 AU and 5.30 ± 2.040 AU, respectively. CF had a Tissue/RBC ratio of 3.06 ± 0.640 AU, and healthy subjects had a Tissue/RBC ratio of 2.66 ± 0.448 AU (*p* < 0.001). Multiple comparisons among healthy subjects and those with diseases were statistically significant (Figure 3, Table 1).

**Figure 4:**
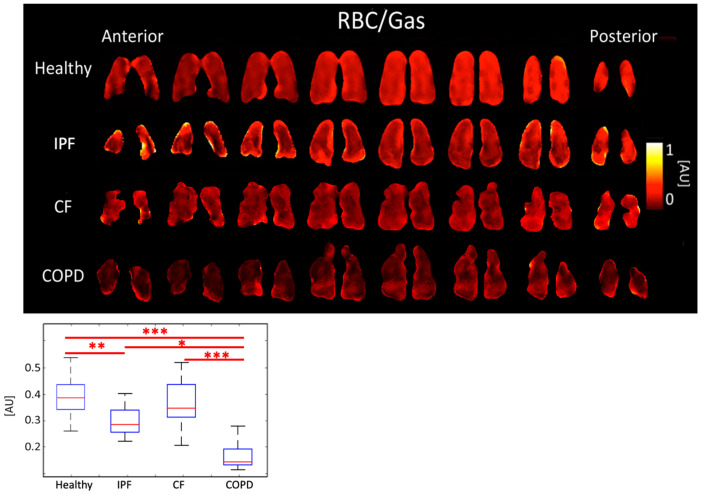
RBC/Gas ratio maps (**top**) and boxplots (**bottom**) for each disease type. (*p* < 0.001 *=* ***, *p* < 0.01 *=* **, *p* < 0.5 *=* *).

COPD subjects had the lowest RBC/gas ratio, 0.15 ± 0.068 AU. The RBC/Gas ratio for CF was 0.35 ± 0.094 AU, IPF was 0.28 ± 0.061 AU, and healthy was 0.39 ± 0.079 AU (*p* < 0.001). The differences between healthy and COPD, and between CF and COPD subjects were most significant (*p* < 0.001). The difference between healthy and IPF was also significant (*p* < 0.01) (Figure 4, Table 1).

**Figure 5:**
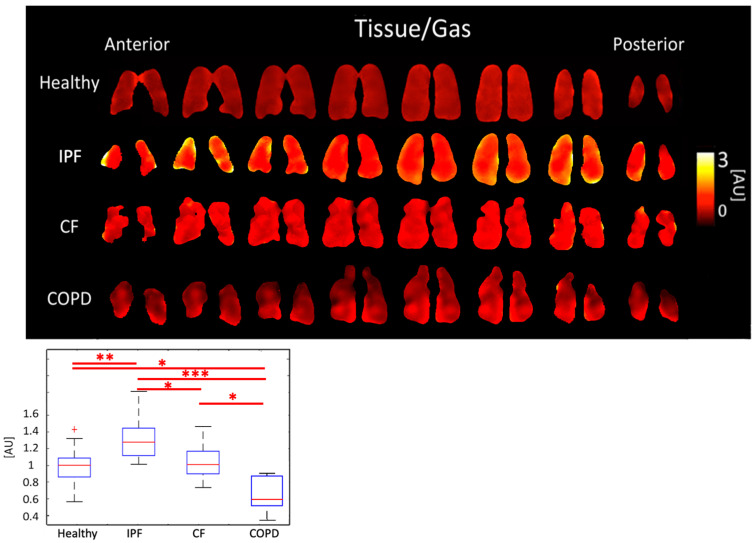
Tissue/Gas ratio maps (**top**) and boxplots (**bottom**) for each disease type. (*p* < 0.001 *=* ***, *p* < 0.01 *=* **, *p* < 0.5 *=* *, ^+^ outlier).

IPF subjects had the highest Tissue/Gas ratio, 1.31 ± 0.259 AU, and COPD subjects had the lowest, 0.66 ± 0.220 AU. Meanwhile, healthy and CF subjects had similar ratios of 0.99 ± 0.196 AU and 1.02 ± 0.196, respectively (*p* < 0.001). The differences between healthy and IPF (*p* < 0.01), and CF and COPD (*p* < 0.001) were significant (Figure 5, Table 1).

### 3.3. Comparison of T2*

**Figure 6:**
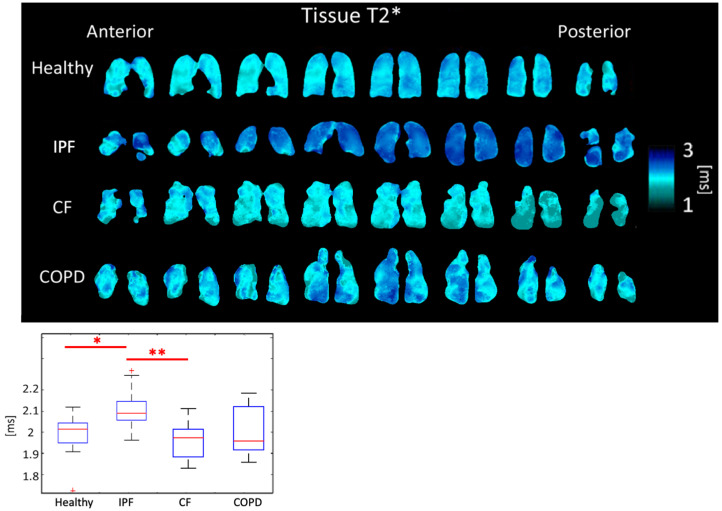
Tissue T2* maps (**top**) and boxplots (**bottom**) for each disease type. (*p* < 0.01 *=* **, *p* < 0.5 *=* *, ^+^ outlier).

Tissue T2* was 2.12 ± 0.093 ms for IPF subjects, 1.97 ± 0.084 ms for CF subjects, 2.02 ± 0.131 ms for COPD subjects, and 2.00 ± 0.089 ms for healthy subjects (*p* < 0.01). Differences between IPF and CF (*p* < 0.01) and between healthy and IPF subjects (*p* < 0.05) were significant (Figure 6, Table 1).

**Figure 7:**
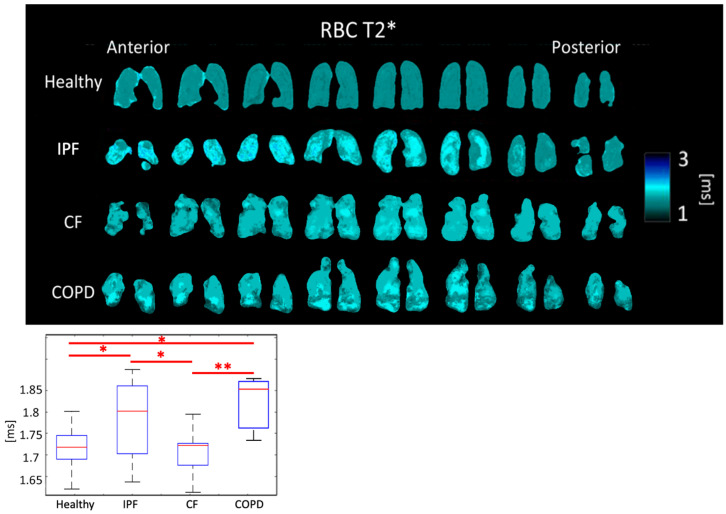
RBC T2* maps (**top**) and boxplots (**bottom**) for each disease type. (*p* < 0.01 *=* **, *p* < 0.5 *=* *).

RBC T2* was 1.79 ± 0.086 ms for IPF subjects, 1.71 ± 0.053 ms for CF subjects, 1.81 ± 0.062 ms for COPD subjects, and 1.71 ± 0.040 ms for healthy subjects (*p* < 0.01). The differences between healthy and IPF, IPF and CF, and CF and COPD were all significant (*p* < 0.05) (Figure 7, Table 1).

### 3.4. Comparison of Chemical Shifts

**Figure 8:**
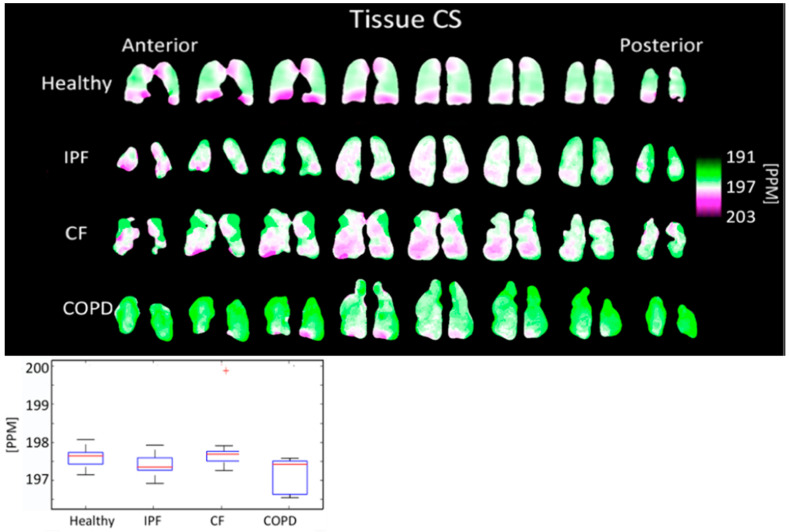
Tissue chemical shift maps (**top**) and boxplots (**bottom**) for each disease type. The color bar corresponds to the location of the geometric center of the tissue peak. Results are not significantly different. (^+^ outlier).

The tissue peak chemical shift was 197.48 ± 0.292 PPM for IPF subjects, 197.87 ± 0.655 PPM for CF subjects, 197.28 ± 0.434 PPM for COPD subjects, and 197.69 ± 0.227 PPM for healthy subjects. The tissue peak chemical shift was not significantly different (*p* > 0.05) between any pair of disease types (Figure 8, Table 1).

**Figure 9:**
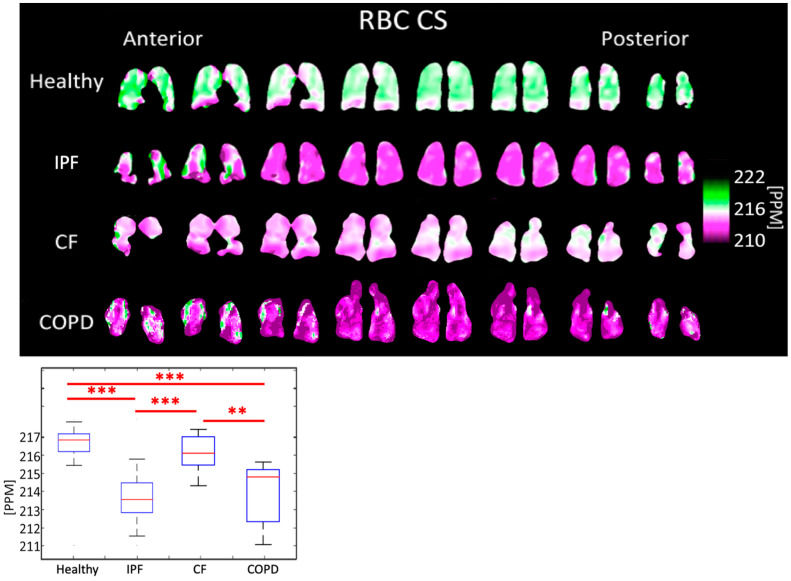
RBC chemical shift maps (**top**) and boxplots (**bottom**) for each disease type. The color bar corresponds to the location of the geometric center of the RBC peak. (*p* < 0.001 *=* ***, *p* < 0.01 *=* **).

The RBC peak chemical shift was 213.49 ± 1.254 PPM for IPF subjects, 215.99 ± 0.910 PPM for CF subjects, 213.74 ± 1.872 PPM for COPD subjects, and 216.60 ± 0.645 PPM for healthy subjects (*p* < 0.001). The differences between healthy and IPF, IPF and CF, healthy and COPD, and CF and COPD were all highly significant (*p* < 0.001) (Figure 9, Table 1).

The separation between RBC and tissue peaks was 16.13 ± 1.208 PPM for IPF subjects, 18.34 ± 0.859 PPM for CF subjects, 16.34 ± 1.884 PPM for COPD subjects, and 18.90 ± 0.628 PPM for healthy subjects (*p* < 0.001). The differences between healthy and IPF, IPF and CF, and healthy and COPD were significant (*p* < 0.001), as well as between CF and COPD (*p* < 0.01) (Table 1).

### 3.5. Correlation with Spirometry

3D-SBCSI data was correlated with spirometry results, FEV1 predicted and FVC predicted, for each subject. The strongest relationships were between FVC predicted and RBC chemical shift (R *=* 0.67) and between FEV1 predicted and RBC/Gas ratio (R *=* 0.67).

### 3.6. Repeatability

For CF and healthy subjects, two 3D-SBCSI acquisitions were taken less than one hour apart. For CF subjects, the average difference between acquisitions for the peak ratios were: Tissue/RBC ratio 7.8 ± 7.99%, Tissue/Gas 9.3 ± 7.37%, and RBC/Gas 13.8 ± 14.93%. The average difference for Tissue T2* was 1.0 ± 0.82% and for RBC T2* was 1.87 ± 1.80%. The average difference for the chemical shifts were: Tissue CS 0.1 ± 0.33%, RBC CS 0.1 ± 0.14%, and RBC-Tissue CS 1.3 ± 0.61.

For healthy subjects, the average difference between acquisitions for the peak ratios were 3.2 ± 2.56% for Tissue/RBC ratio, 2.5 ± 3.03% for Tissue/Gas, and 5.3 ± 4.30% for RBC/Gas. The average difference for Tissue T2* was 0.8 ± 0.69% and for RBC T2* was 2.3 ± 2.68%. The average difference for the chemical shifts were: Tissue CS 0.02 ± 0.02%, RBC CS 0.1 ± 0.04%, and RBC-Tissue CS 0.6 ± 0.46%.

CF subjects had a larger difference between acquisitions compared to healthy subjects, but this may be due to the fact that CF patients had greater variation in the architecture of their lungs than healthy subjects. Results were not expected to be exactly the same due to potential differences between breath-holds [31].

## 4. Discussion

To demonstrate the power of 3D-SBCSI hyperpolarized Xe-29 MRI to differentiate among lung diseases, 45 subjects with either CF, COPD, IPF, or healthy lungs were imaged using a ~7 s breath-hold without any complications. Additionally, proton and ventilation images were taken for each subject and overlaid to map and analyze the level of lung ventilation in each disease group. Overall, the results demonstrated differences in ventilation between the disease groups that were expected based on the underlying pathology of each disease. Peak ratios and T2* were also able to elucidate differences between CF, COPD, IPF, and healthy subjects. Subjects underwent spirometry as well, and the results correlated with 3D-SBCSI results.

### 4.1. Comparison of Ventilation Images

While CF and healthy subjects had no statistically significant differences in 3D-SBCSI parameters, differences were seen in ventilation images. CF subjects had more ventilation defects (no ventilation) than IPF and healthy subjects, which likely corresponded to the locations of mucus plugs in the lungs. Our results showing increased ventilation defects in CF are consistent with the xenon MRI in CF literature [32].

In IPF, the large areas of hypoventilation rather than of no ventilation were expected as IPF is not an obstructive lung disease (Figure 1 and Figure 2).

The reduced volume of normally ventilated regions was significant in IPF, CF, and COPD in comparison with healthy subjects. The segmented ventilation images conveyed which regions of the lungs experienced poor ventilation.

### 4.2. Comparison of Peak Ratios

The average Tissue/RBC ratio was higher in IPF and COPD subjects than in CF and healthy subjects (Figure 3) because of impaired gas exchange from tissue damage in IPF and COPD. Additionally, IPF and COPD subjects had more heterogeneous Tissue/RBC ratios throughout the lungs than healthy and CF subjects; notably, in IPF subjects, the periphery of the lungs had elevated Tissue/RBC ratios, which is consistent with the distribution of abnormalities observed using other imaging modalities [33]. This suggests localized, severely compromised gas exchange ability, which could be used as a marker for tracking disease progression in these subjects. Regions of high Tissue/RBC ratios may correspond to locations where ventilation and perfusion are mismatched, which is expected in both COPD and IPF.

IPF and COPD subjects also had lower RBC/Gas ratios than healthy and CF subjects. This was expected because IPF and COPD subjects have impaired gas transfer between parenchymal tissue and RBCs. COPD subjects had even lower RBC/Gas ratios than IPF subjects because not only was gas transfer impaired but there was also less tissue and fewer blood vessels for the gas to transfer to RBCs. The RBC/Gas ratio in CF subjects was expected to be the same or slightly lower than in healthy subjects because tissue is generally healthy in this population, apart from mucus plugging, so gas transfer to RBCs is relatively unrestricted (Figure 4).

IPF and COPD subjects had significantly different Tissue/Gas ratios compared to healthy subjects. Given that IPF is characterized by a thicker, fibrotic parenchyma [34], more 129-Xe would dissolve into lung tissue resulting in a higher Tissue/Gas ratio. COPD is characterized by the destruction of parenchyma, so, as expected, little 129-Xe would dissolve in tissue. These results, seen on Figure 5, are consistent with findings by Wang et al. [35]. CF is characterized by airway obstruction rather than tissue defects. Thus, the Tissue/Gas ratio was not and would not be expected to be significantly different from healthy subjects.

The only significantly different 3D-SBCSI parameters between IPF and COPD subjects were Tissue/Gas and RBC/Gas ratios (*p* < 0.001). Although both are tissue diseases, the parenchyma is affected differently in IPF and COPD, which affects how much gas dissolves into RBCs. Despite differences in overall tissue or RBC dissolved-phase gas, the Tissue/RBC ratio was not significantly different between IPF and COPD subjects.

### 4.3. Comparison of T2*

Physiological reasons for changes in the Tissue and RBC T2* have not yet been clearly identified or understood. However, Wolber et al. hypothesized that changes in Tissue T2* are caused by the increasingly frequent exchange of xenon between the plasma and RBCs [36]. The average Tissue T2* and RBC T2* were significantly longer in IPF subjects than in healthy subjects. Tissue T2* was likely longer due to tissue thickening in the lungs of IPF subjects leading to less interactions due to increased distance between air/tissue interfaces. CF subjects had shorter Tissue T2* times than each of the other groups. CF subjects are also known to have elevated ferritin levels that create local susceptibilities, accelerating decay time and shortening T2* [37]. As expected, COPD subjects had a similar Tissue T2* to healthy subjects (Figure 6).

RBC T2* was longer in IPF and COPD subjects perhaps due to the lower blood oxygenation. CF subjects do not have impaired gas exchange nor do have thick, fibrotic tissues, so their RBC T2* was expected to be similar to healthy subjects, which it was the case.

Regional heterogeneities in RBC T2* maps corresponded well with heterogeneities in the Tissue/RBC maps (Figure 3 and Figure 7). However, global values for RBC T2* and Tissue/RBC were not significantly correlated (R = 0.40), which indicates that there could be several interdependent causes of impaired gas exchange (higher Tissue/RBC ratios).

### 4.4. Comparison of Chemical Shifts

The tissue center did not shift significantly in any subjects (Figure 8). The RBC center shifted by an average of 2.86 PPM in COPD subjects, 3.11 PPM in IPF subjects, and 0.61 PPM in CF subjects, relative to healthy subjects (Figure 9). In all groups, the RBC peak moved closer to the tissue peak, reducing the separation between the peaks by 2.35 PPM in COPD subjects, 2.77 PPM in IPF subjects, and 0.56 PPM in CF subjects, relative to healthy subjects.

Previous studies found that RBC chemical shift was approximately 2 PPM lower in IPF subjects than in healthy subjects while the tissue chemical shift was not significantly different [38]. Norquay et al. found that as the blood oxygenation level increased the RBC peak shifted further from the stationary gas peak [39]. Based on this, a decrease in the RBC chemical shift was expected in hypoxic IPF and COPD subjects [40,41]. These studies were completed using whole-lung HP Xe-129 spectroscopy, whereas our work presents comparable results on a regional level.

### 4.5. Correlation with Spirometry

The 3D-SBCSI results were analyzed along with spirometry results to further understand 3D-SBCSI parameters. However, the two probe fundamentally different phenomena: 3D-SBCSI probes gas exchange regionally, while spirometry probes whole-lung ventilation. The correlation between FVC predicted and Tissue/RBC for healthy and COPD subjects was very weak, as was the correlation between FVC predicted and RBC chemical shift. 3D-SBCSI data was able to separate subjects more distinctly than spirometry. Eleven subjects had an FVC predicted between 75–85% (full range for all subjects was 31–136%) but their RBC chemical shifts ranged from 212–217 PPM (full range for all subjects was 211.0–217.8 PPM).

This indicated that Tissue/RBC and RBC CS were more sensitive parameters than FVC predicted or FEV1 predicted and are capable of distinguishing between subjects with similar FVC predicted values among the same disease. These 3D-SBCSI parameters may be sensitive to subtle changes caused by disease phenotype or differences in pathological alterations in lung structure that affect gas exchange and ventilation. For example, there were four subjects with FVC predicted of 94–95% but Tissue/RBC ranged from 2.11 to 3.04 and RBC CS ranged from 215.95 to 217.11 for the same subjects. In addition, six subjects had an FVC predicted of 76–77%, and Tissue/RBC ranged from 3.27 to 5.00 for these subjects. The differences may be a result of disease severity and Xe-129 MRI seems to provide better differentiation between patients who have similar lung function and thus may provide a more objective measure of disease, which would be a great tool for providing more personalized treatment.

### 4.6. Limitations

Study limitations included small sample size and no differentiation by severity within IPF and COPD subjects. Our healthy volunteers tended to be closer to the age range of CF participants. While a limitation, this is also consistent with previous xenon MRI literature. Healthy and CF subjects were much younger than IPF and COPD subjects due to the differing disease populations which for CF tends to be a pediatric disease while COPD and IPF only manifests later in life.

## 5. Conclusions

The results of this study indicate that 3D-SBCSI is sensitive to the physiology of lung diseases and can therefore be used to help differentiate among healthy, IPF, CF, and COPD lung disease types. This method also provides additional MRI based markers that may reflect the underlying lung physiology, like voxel based full Xe-129 gas spectra, multiple lung compartment T2*, and chemical shift, which no other current techniques can offer. All this regional information combined may be useful for monitoring disease progression on a regional level as well as for characterizing disease phenotypes and co-morbidities in the future.

## Figures and Tables

**Table 1:** Averages and standard deviations for subjects and computed parameters for each disease type.

		Healthy	IPF	CF	COPD
	**Age**	28 ± 9.8	66 ± 11.6	24 ± 8.7	64 ± 11.8
	**M/F**	5/11	8/3	3/10	4/1
	**FVC Predicted [%]**	103 ± 8.8	66 ± 15.0	92 ± 19.6	91 ± 6.3
	**FEV1 Predicted [%]**	99 ± 7.6	67 ± 14.7	76 ± 23.6	55 ± 20.9
**Ventilation**	**No Ventilation [%]**	0.4 ± 0.50	2.2 ± 0.91	14.1 ± 8.37	20.3 ± 11.25
	**Hypoventilation [%]**	9.2 ± 6.96	25.8 ± 6.31	25.0 ± 6.41	39.1 ± 2.23
	**Normal Ventilation [%]**	90.4 ± 7.37	72.0 ± 6.44	60.9 ± 13.86	40.6 ± 9.14
**Whole-Lung CSI Averages**	**Tissue/RBC [AU]**	2.66 ± 0.448	4.71 ± 0.807	3.06 ± 0.640	5.30 ± 2.040
	**RBC/Gas [AU]**	0.39 ± 0.079	0.28 ± 0.061	0.35 ± 0.094	0.15 ± 0.068
	**Tissue/Gas [AU]**	0.99 ± 0.196	1.31 ± 0.259	1.02 ± 0.196	0.66 ± 0.220
	Tissue T2* [ms]	2.00 ± 0.089	2.12 ± 0.093	1.97 ± 0.084	2.02 ± 0.131
	RBC T2* [ms]	1.72 ± 0.040	1.79 ± 0.086	1.71 ± 0.053	1.82 ± 0.061
	**Tissue CS [PPM]**	197.69 ± 0.227	197.48 ± 0.292	197.87 ± 0.655	197.28 ± 0.434
	**RBC CS [PPM]**	216.60 ± 0.645	213.49 ± 1.254	215.99 ± 0.910	213.74 ± 1.872
	**RBC-Tissue CS [PPM]**	18.90 ± 0.627	16.13 ± 1.21	18.34 ± 0.859	16.55 ± 1.698

## Data Availability

Not applicable.
